# Prediction of cardiac events using fully automated GLS and BNP titers in patients with known or suspected heart failure

**DOI:** 10.1371/journal.pone.0234294

**Published:** 2020-06-15

**Authors:** Kyoko Otani, Yukie Higa, Tetsuji Kitano, Yosuke Nabeshima, Masaaki Takeuchi

**Affiliations:** 1 Department of Laboratory and Transfusion Medicine, Hospital of University of Occupational and Environmental Health, School of Medicine, Kitakyushu, Japan; 2 Second Department of Internal Medicine, University of Occupational and Environmental Health, School of Medicine, Kitakyushu, Japan; International University of Health and Welfare, School of Medicine, JAPAN

## Abstract

**Background:**

Although global longitudinal strain (GLS) measurements provide useful predictive information, measurement variability is still a major concern. We sought to determine whether fully automated GLS measurements could predict future cardiac events in patients with known or suspected heart failure (HF).

**Methods:**

GLS was measured using fully automated 2D speckle tracking analysis software (AutoStrain, TomTec) in 3,150 subjects who had undergone clinically indicated brain natriuretic peptide (BNP) assays and echocardiographic examinations. Among 1,514 patients in the derivation cohort, optimal cut-off values of BNP and GLS for cardiac death (CD) and major adverse cardiovascular events (MACEs) were determined using survival classification and regression tree (CART) analysis. The remaining 1,636 patients, comprising the validation cohort, were stratified into subgroups according to predefined cut-off values, and survival curves were compared.

**Results:**

Survival CART analysis selected GLS with cut-off values of 6.2% and 14.0% for predicting CD. GLS of 6.9% and 13.9% and BNP of 83.2 pg/mL and 206.3 pg/mL were selected for predicting MACEs. For simplicity, we defined GLS of 7% and 14% and BNP of 100 pg/mL and 200 pg/mL as cut-off values. These cut-off values stratify high-risk patients in the validation cohort with known or suspected HF for both CD and MACEs.

**Conclusions:**

In addition to BNP, fully automated GLS measurements provide prognostic information for patients with known or suspected HF, and this approach facilitates clinical work flow.

## Introduction

Heart failure (HF) is a global public health concern because it is a major cause of mortality and morbidity, especially in countries with aging populations [1, 2]. Repeated hospitalization is costly and impacts national healthcare budgets [3], so it is important to accurately evaluate the prognosis of HF patients while conserving medical resources.

Two-dimensional (2D) transthoracic echocardiography (TTE) is a routine method of choice for patients with known or suspected HF. HF patients are classified by left ventricular (LV) ejection fraction (EF) into HF with preserved EF (HFpEF), HF with mid-range EF (HFmrEF), and HF with reduced EF (HFrEF) [4]. Although LVEF is a critical indicator of cardiac function, LVEF is not capable of detecting latent LV dysfunction. Previous studies have demonstrated that myocardial strain, based on 2D speckle tracking analysis, is simple and feasible, and constitutes a strong, independent prognostic factor for HF patient outcomes, independent of LVEF [5–8]. Although global longitudinal strain (GLS) measurements are more reproducible than conventional LVEF measurements, observer variability can still be a confounding factor [9, 10]. The advent of fully automated speckle tracking software may overcome this problem. Brain natriuretic peptide (BNP) is a clinically powerful biomarker for predicting recurrent HF hospitalizations and death [11–13]. We hypothesized that fully automated GLS measurements could potentially predict outcomes in HF patients.

The aim of this study was to investigate the enhanced prognostic value of fully automated GLS measurements over BNP measurements and other predictors in patients with known or suspected HF.

## Methods

### Study design and ethics

This was a retrospective, single-center observational study conducted in Japan. We selected a total of 3,150 consecutive subjects (68±15 years, 2,615 men) who had undergone both BNP measurements and 2D TTE examinations within a 1-week interval that were ordered at the clinical discretion of each attending physician for patients with known or suspected HF from January 2015 to December 2016. Of the 3,150 echocardiography examinations, 82 examinations were excluded from the analysis because of a lack of apical images, and an additional 655 examinations were excluded because they were repeat examinations of the same patient, resulting in a total of 2,413 patients in the final analysis. In order to determine optimal cut-off values of BNP and fully automated GLS for predicting future cardiac death (CD) and major adverse cardiovascular events (MACEs), and to verify the usefulness of these prognostic values, the final cohort was divided into a derivation group (1,157 patients who were selected during 2015) and a validation group (1,256 patients who were selected during 2016).

The study protocol was approved by the Institutional Review Board at the University of Occupational and Environmental Health, School of Medicine. The requirement for informed consent was waived due to the retrospective nature of the study.

### Echocardiography

**Fig 1:**
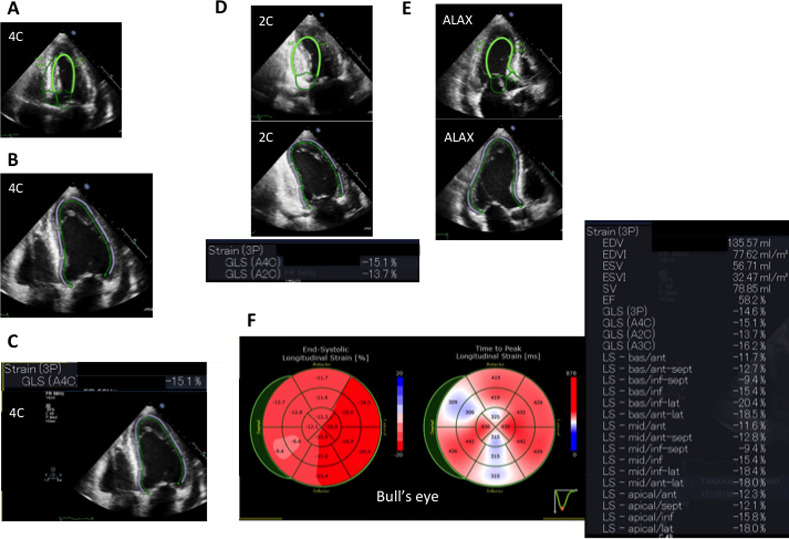
Step-by step approach to left ventricular (LV) volume and function measurements. A: Manual registration of apical 4-chamber view at end-diastole. B: Automatic LV endocardial border tracing (green line) at end-diastole in apical 4-chamber view. C: 2D speckle tracking analysis throughout the cardiac cycle, and a GLS of 4-chamber view was determined. D: The same procedures on apical 2-chamber view. E: The same procedures on long-axis view. F: Displayed bull’s eye, LV volumes, LV ejection fraction (EF) and global longitudinal strain (GLS).

2D TTE was performed using a commercially available ultrasound machine and transducer (iE33; Philips Medical System, Andover, MA or Vivid7; GE Healthcare, Horten, Norway). For each patient, after manual registration of an apical 4-chamber view (Fig 1A), the LV endocardial border at end-diastole was automatically determined using fully automated speckle tracking software (AutoSTRAIN; TomTec Imaging Systems, Unterschleissheim, Germany) (Fig 1B). The software performed 2D speckle tracking analysis throughout the cardiac cycle, from which GLS of the apical 4-chamber view was determined (Fig 1C). Subsequently, the same procedures were performed on apical 2-chamber (Fig 1D) and long-axis views (Fig 1E). Finally, GLS, bull’s eye plots of regional longitudinal strain, LV end-diastolic volume (EDV), LV end-systolic volume, and LVEF by the tri-plane method of disks were obtained (Fig 1F). We used results from fully automated analysis, and did not manually correct LV endocardial borders in the patients.

### Blood examination test

BNP levels were measured in fresh samples using a commercially available assay (Centaur XP, Siemens Inc., Tokyo, Japan) at the time of the medical examination. The following parameters measured on the same day as the BNP measurements were also collected from the electric medical records: sodium, creatinine and hemoglobin that are known to be significant predictors in HF [14, 15].

### Follow-up

Follow-up information was obtained from clinical visits or telephone interviews. The primary endpoint was CD, and the secondary endpoint was a composite of cardiac events, including CD, non-fatal myocardial infarction, ventricular fibrillation/tachycardia, and HF requiring hospitalization. Total follow-up time was calculated as the elapsed time until a subsequent event occurred or until the last follow-up time, in cases that had no endpoint.

### Reproducibility

The variability of GLS measurements was assessed by measuring the same images three times in 10 randomly selected patients. Analysis times from selecting images of 3 apical views to obtaining the final results were also measured in the same 10 patients who were included in reproducibility analysis. Test-retest variability was assessed by another apical 3 images that were acquired by two different sonographers during the same examinations in 30 randomly selected patients.

### Statistical analyses

Continuous data are presented as means ± SD for normally distributed data or medians with interquartile ranges (IQR: 25^th^ percentile to 75^th^ percentile) for skewed distributions. Categorical data are presented as absolute values or percentages. The statistical significance of differences between the means of two groups was assessed with Student’s t-tests, and the significance of differences in proportions was evaluated using the chi-squared statistic. Cox proportional hazard regression analysis was used to calculate hazard ratios (HRs) and 95% confidence intervals (95% CIs). We conducted survival classification regression-tree (CART) analysis to identify preferential markers and optimal cut-off values for prediction of CD and MACEs. Kaplan-Meier survival analysis was performed and group comparisons were conducted using the log-rank test. Incremental improvement in prognostic values was also assessed using the nested regression model and net reclassification improvement (NRI). Reclassification analysis was performed to assess NRI when adding GLS to the significant anthropometric factors, blood examination parameters and fully automated echocardiographic predictors derived from univariable Cox proportional hazard model in the derivation cohort. P <0.05 was considered statistically significant. All statistical analyses were performed with commercial software (JMP version 13.1, SAS Institute Inc., Cary, NC; SPSS version 24, Chicago, IL; R version 3.4.3, R Foundation for Statistical Computing, Vienna, Austria; and Prism version 7.0d, GraphPad Software Inc., San Diego, CA).

## Results

Forty patients (4%) in the derivation group and 78 patients (7%) in the validation group were excluded from the analysis due to erroneous LV contour determination. Clinical characteristics of the derivation and validation groups are shown in Table 1. There were no significant differences between the two groups except for body surface area (BSA).

**Table 1:** Characteristics of the study population.

Variable	derivation group	validation group	p
Number	1117	1178	
Age (year)	68±14	69±13	0.0641
Men/women	602/515	676/502	0.0924
BMI (kg/m^2^)	22.7±4.0	22.9±3.9	0.3588
BSA (m^2^)	1.58±0.20	1.59±0.20	0.0286
SBP (mmHg)	138±24	136±25	0.1649
DBP (mmHg)	77±13	77±14	0.2792
NYHA class l/ll/lll/lV	419/517/107/74	430/542/124/82	0.8559
Hypertension (%)	672 (60)	683 (58)	0.2881
Diabetes (%)	321 (29)	339 (29)	0.9832
Dyslipidemia (%)	366 (33)	401 (34)	0.5177
Atrial fibrillation (%)	227 (20)	236 (20)	0.8634
End-stage renal failure (%)	70 (6)	69 (6)	0.6811
Chronic obstructive pulmonary disease (%)	63 (6)	81 (7)	0.2223
Smoking yes/quit/no	166/398/519	186/438/529	0.6203
Ischemic heart disease (%)	267 (24)	285 (24)	0.8708
Valvular heart disease (%)	146 (13)	142 (12)	0.4625
Dilated cardiomyopathy (%)	74 (7)	58 (5)	0.0802
Secondary cardiomyopathy (%)	91 (8)	89 (8)	0.5982
Hypertrophic cardiomyopathy (%)	22 (2)	23 (2)	0.9764
Beta-blockers	313 (28)	326 (28)	0.8527
ACE/ARB	512 (46)	523 (44)	0.4884
BNP (pg/mL)	65 (IQR; 24 to 212)	66 (IQR; 25 to 205)	0.9886
Sodium (mEq/L)	139±3	139±4	0.8430
Creatinine (mg/dL)	0.89 (IQR; 0.7 to 1.38)	0.88 (IQR; 0.69 to 1.25)	0.2987
Hemoglobin (g/dL)	12.3±2.2	12.4±2.2	0.2259
LVEDVI (mL/m2)	63±19	62±19	0.4295
LVESVI (mL/m2)	35±18	35±17	0.3978
LVEF (%)	46±12	46±12	0.9207
GLS (%)	14.4±5.2	14.6±5.2	0.3625

Continuous data are expressed as mean±standard deviation or median and interquartile range (IQR). Categorical data are presented as the absolute value and percentage.

ACE, angiotensin-converting enzyme inhibitors; ARB, angiotensin receptor blockers; BMI, body mass index; BNP, B-type natriuretic peptide; BSA, body surface area; DBP, diastolic blood pressure; GLS, global longitudinal strain; LVEDVI, left ventricular end-diastolic volume index; LVEF, left ventricular ejection fraction; LVESVI, left ventricular end-systolic volume index; NYHA, New York Heart Association; SBP, systolic blood pressure.

### Determination of cut-off values for predicting cardiac death and MACE in the derivation cohort

Follow-up data could not be obtained for 74 patients (7%) in the derivation group because of loss of contact. During a median follow-up of 32.3 months (IQR: 20.0–39.4 months), 52 patients reached the primary endpoint, while 134 reached secondary endpoints, including 52 CDs, 76 HFs requiring hospitalization, 5 non-fatal myocardial infarctions, and 1 case of ventricular tachyarrhythmia.

Tables 2 and 3 depict clinical and echocardiography variables between patients with or without events and the univariable Cox proportional hazard models in the derivation group. Univariate analysis revealed that age, sex, systolic blood pressure (SBP), BNP, sodium, creatinine, New York Heart Association (NYHA) classification, LV end-diastolic volume index (LVEDVI), LVEF, and GLS were significantly associated with CD (Table 2), and showed that age, BNP, sodium, creatinine, hemoglobin, NYHA classification, LVEDVI, LVEF and GLS were significantly associated with MACEs (Table 3).

**Table 2:** Univariable cox proportional analyses for cardiac death in the derivation group.

	cardiac death (+)	cardiac death (-)		Univariable analysis		
	(n = 52)	(n = 991)	p	HR	95% CI	p
Age (y)	74±13	68±14	0.0037	1.042	1.017–1.070	0.0006
Sex (male/female)	35/17	536/455	0.0619	0.555	0.304–0.976	0.0407
SBP (mmHg)	130±25	138±24	0.0215	0.985	0.973–0.997	0.0170
BMI (kg/m^2^)	23.1±4.6	22.7±4.0	0.4730	1.015	0.948–1.077	0.6558
BNP (pg/mL)	705±807	204±459	<0.0001	1.001	1.000–1.001	<0.0001
Sodium (mEq/L)	138±3	139±3	0.0216	0.898	0.845–0.964	0.0044
Creatinine (mg/dL)	3.14±3.16	1.75±2.39	<0.0001	1.147	1.064–1.222	0.0008
Hemoglobin (g/dL)	11.9±2.3	12.4±2.2	0.1068	0.883	0.779–1.002	0.0542
NYHA (I,II/III,IV)	19/33	852/139	<0.0001	10.358	5.948–18.564	<0.0001
LVEDVI (mL/m^2^)	76.1±25.0	62.4±19.0	<0.0001	1.024	1.013–1.033	<0.0001
LVEF (%)	33.3±13.1	46.6±12.0	<0.0001	0.928	0.909–0.946	<0.0001
GLS (%)	8.6±5.1	14.7±5.0	<0.0001	0.806	0.764–0.849	<0.0001

BMI, body mass index; BNP, B-type natriuretic peptide; CI, confidence interval; GLS, global longitudinal strain; HR, hazard ratio; LVEDVI, left ventricular end-diastolic volume index; LVEF, left ventricular ejection fraction; NYHA, New York Heart Association; SBP, systolic blood pressure.

**Table 3:** Univariable cox proportional analyses for MACEs in the derivation group.

	MACEs (+)	MACEs (-)		Univariable analysis		
	(n = 134)	(n = 909)	p	HR	95% CI	p
Age (y)	73±12	67±14	<0.0001	1.042	1.026–1.059	<0.0001
Sex (male/female)	80/54	491/418	0.2170	0.777	0.547–1.094	0.1484
SBP (mmHg)	134±26	138±24	0.0848	0.993	0.985–1.000	0.0545
BMI (kg/m^2^)	22.7±3.8	22.7±4.1	0.9430	0.993	0.951–1.034	0.7449
BNP (pg/mL)	577±739	177±423	<0.0001	1.001	1.001–1.001	<0.0001
Sodium (mEq/L)	139±3	139±3	0.1794	0.938	0.895–0.987	0.0155
Creatinine (mg/dL)	2.83±2.97	1.66±2.33	<0.0001	1.123	1.070–1.173	<0.0001
Hemoglobin (g/dL)	11.6±2.2	12.5±2.2	<0.0001	0.823	0.761–0.891	<0.0001
NYHA (I,II/III,IV)	70/64	801/108	<0.0001	6.156	4.374–8.649	<0.0001
LVEDVI (mL/m^2^)	74.8±24.7	61.4±18.1	<0.0001	1.023	1.016–1.029	<0.0001
LVEF (%)	36.2±13.3	47.3±11.5	<0.0001	0.940	0.928–0.951	<0.0001
GLS (%)	10.1±5.1	15.0±4.9	<0.0001	0.843	0.816–0.870	<0.0001

BMI, body mass index; BNP, B-type natriuretic peptide; CI, confidence interval; GLS, global longitudinal strain; HR, hazard ratio; LVEDVI, left ventricular end-diastolic volume index; LVEF, left ventricular ejection fraction; MACEs, major adverse cardiovascular events; NYHA, New York Heart Association; SBP, systolic blood pressure.

**Fig 2:**
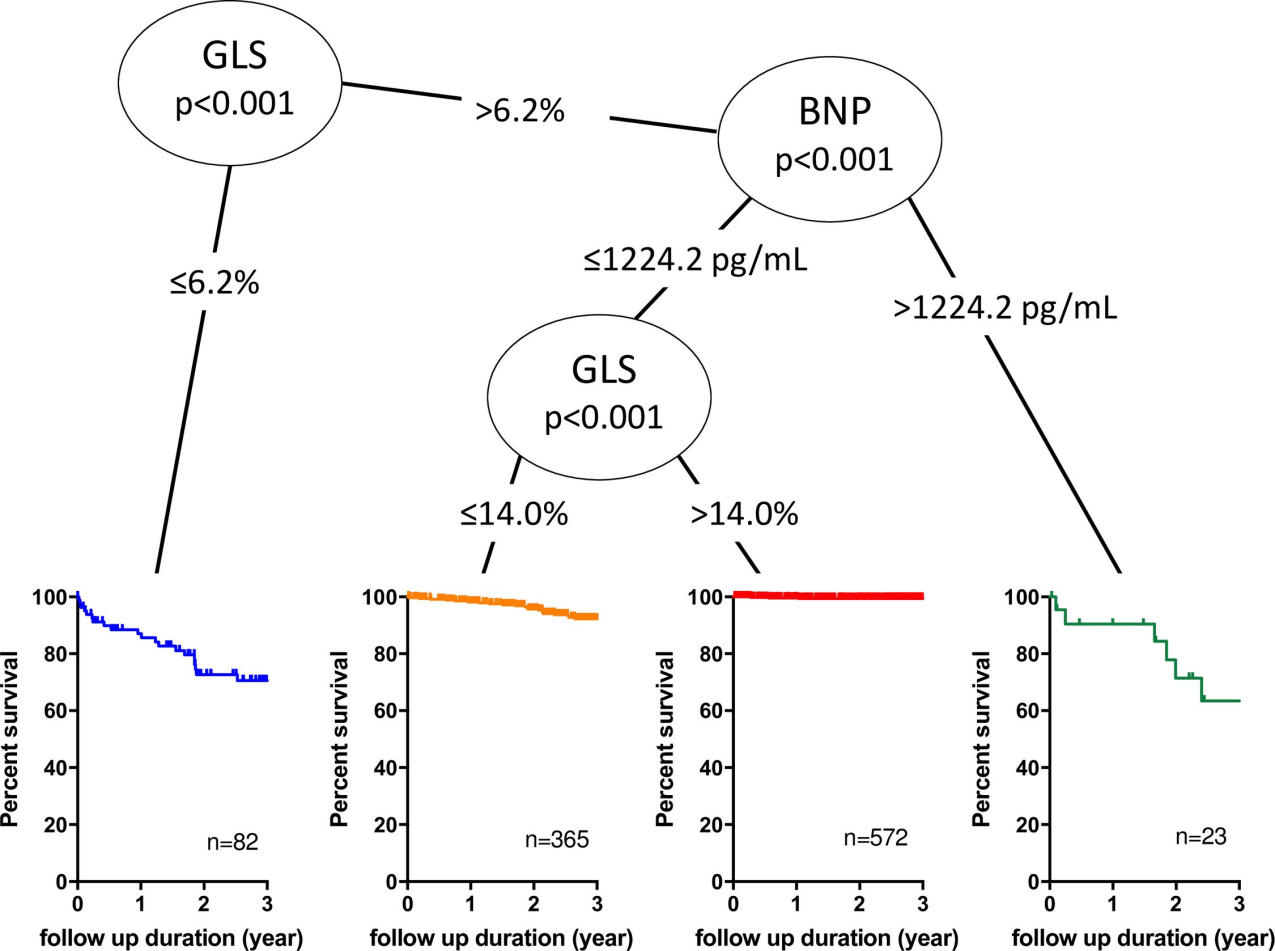
Optimal cut-off values of BNP and GLS, and Kaplan-Meier survival curves for predicting future cardiac death (CD) in the derivation group. Survival CART analysis identified optimal BNP and GLS cut-off values to partition the risk of CD. Colored lines represent the survival curve for CD in each subset, classified using BNP and GLS cut-off values. BNP; B-type natriuretic peptide, GLS; global longitudinal strain, CART; classification and regression trees.

Survival CART analysis, including LVEDVI, LVEF, GLS, and BNP, revealed that a GLS of 6.2%, BNP of 1224.2 pg/mL, and GLS of 14.0% were selected for stratification into 4 groups for CD (Fig 2). Kaplan-Meier survival curves showed that patients who had GLS< 6.2% or patients who had GLS > 6.2% and BNP > 1224.2 pg/mL had worse prognoses, followed by patients with GLS ranging from 6.2% to 14.0%. The best event-free survival curve was observed in patients with GLS > 14.0%. Since the number of patients who had GLS > 6.2% and BNP > 1224.2 pg/mL was very small (n = 23), we determined that BNP of 1224.2 pg/mL was not clinically relevant.

**Fig 3:**
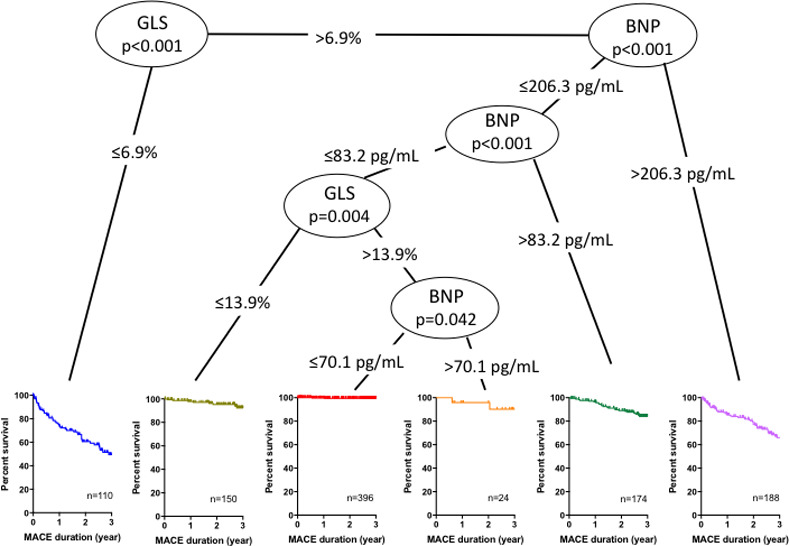
Optimal cut-off values of BNP and GLS, and Kaplan-Meier survival curves for predicting major adverse cardiovascular events (MACEs) in the derivation group. Survival CART analysis identified optimal BNP and GLS cut-off values to partition the risk of MACEs. Colored lines represent the survival curve for MACEs in each subset, classified using BNP and GLS cut-off values. BNP; B-type natriuretic peptide, GLS; global longitudinal strain, CART; classification and regression trees.

Regarding MACEs, CART analysis included the same four parameters, first GLS of 6.9%, followed by BNP of 206.3 pg/mL, BNP of 83.2 pg/mL, GLS of 13.9%, and lastly BNP of 70.1 pg/mL, resulting in 6 distinct groups of patients (Fig 3). Kaplan-Meier survival curves showed that patients who had GLS< 6.9% or patients who had GLS > 6.9% and BNP > 206.3 pg/mL had worse prognoses, followed by patients with GLS > 6.9% and BNP > 83.2 pg/mL. Since the number of patients who had GLS > 13.9% and BNP > 70.1 pg/mL was very small (n = 24), we discarded BNP of 70.1 pg/mL for prognostication, leaving five subgroups.

To make cut-off values easier to remember, we approximated GLS cut-off values of 7% and 14% for predicting CD, resulting in the following three subgroups: Group 1: GLS < 7%; Group 2: GLS of 7% to 14%; Group 3: GLS > 14%. Regarding MACEs, we used GLS of 7% and 14% and BNP of 100 pg/mL and 200 pg/mL to stratify patients into 5 groups for subsequent analysis: Group 1: GLS < 7%; Group 2: GLS ≥ 7% and BNP > 200 pg/mL; Group 3: GLS ≥ 7% and BNP of 100–200 pg/mL; Group 4: GLS of 7% - 14% and BNP < 100 pg/mL; Group 5: GLS > 14% and BNP < 100 pg/mL.

### Validation of these cut-offs

Fifty-three (4%) patients were lost during follow-up in the validation cohort. During a median follow-up of 24.4 months (IQR: 12.6–27.5 months), 51 patients developed CD, while 123 reached secondary endpoints, including 51 CDs, 67 HFs requiring hospitalization, 4 non-fatal myocardial infarctions, and 1 case of ventricular tachyarrhythmia.

**Fig 4:**
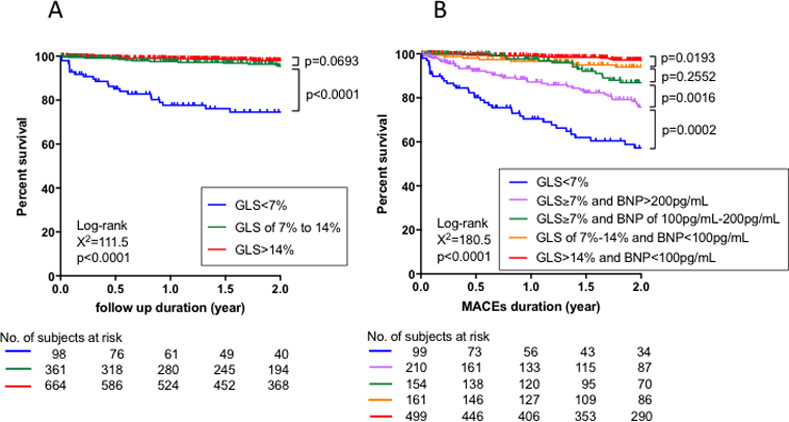
Kaplan−Meier survival analysis for cardiac death (CD; A) and major adverse cardiovascular events (MACEs; B) in the validation group. Each patient was classified into 1 of 3 groups, based upon GLS values from the CART analysis (Fig 4A), while each patient was classified into 1 of 5 groups according to cut-off values for BNP and GLS from the CART analysis (Fig 4B). Colored lines represent survival curves for CD (A) and MACEs (B). BNP; B-type natriuretic peptide, GLS; global longitudinal strain, CART; classification and regression trees.

Fig 4A shows Kaplan-Meier survival analysis for CD among three subgroups. Significantly poorer prognosis was observed in Group 1 (GLS< 7%) compared to Groups 2 and 3. No statistically significant difference in survival was observed between Groups 2 and 3.

Fig 4B shows Kaplan-Meier survival analysis for MACEs among five subgroups. There was a stepwise reduction of MACE-free survival from Group 5 to Group 1, and neighborhood group comparison revealed statistically significant differences in survival, except for the comparison between Groups 3 and 4.

**Fig 5:**
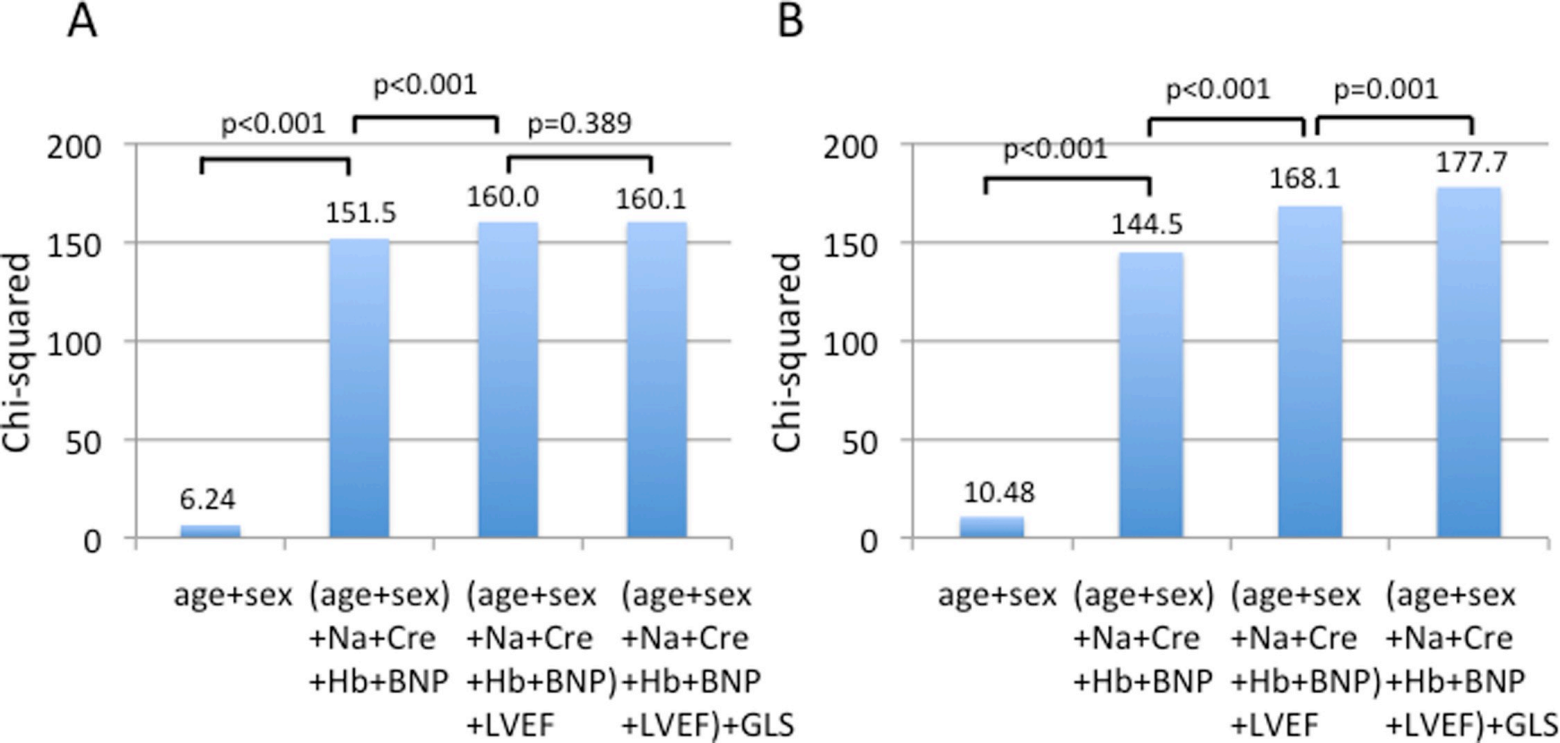
Nested regression model assessing incremental values of blood examination parameters and GLS in the validation group. BNP, sodium, creatinine and hemoglobin afforded significant incremental improvement over the model including age and sex, and LVEF provided significant additional prognostic value over the model including age, sex, BNP, sodium, creatinine and hemoglobin. The further addition of GLS had no incremental value for predicting risk of cardiac death (A) but had a significant incremental value for predicting major adverse cardiovascular events (MACEs; B). BNP; B-type natriuretic peptide, Cre; creatinine, GLS; global longitudinal strain, Hb; hemoglobin, LVEF; left ventricular ejection fraction, Na; sodium.

Fig 5A and 5B show a nested regression model using age, sex, BNP, sodium, creatinine, hemoglobin, LVEF, and GLS in the stepwise analysis for CD and MACEs. For CD, chi-squared values were significantly increased by adding BNP, sodium, creatinine and hemoglobin to the model including age and sex, and were further increased by adding LVEF. However, further addition of GLS to the model had no incremental value. For MACE, sequential addition of BNP, sodium, creatinine and hemoglobin, and LVEF increased chi-square value compared with the chi-square value of the model with age and sex. The addition of GLS further increased chi-square value.

Tables 4 and 5 show reclassification analyses assessing NRI when GLS was added to the significant anthropometric factors (age and sex), blood examination parameters (BNP, sodium, creatinine and hemoglobin), and LVEF. Although addition of GLS resulted in no improvements of prediction model for cardiac death, it resulted in an improved prediction model for MACEs with a significant increase in the continuous NRI of 0.36 (95% CI: 0.17–0.56, p<0.001).

**Table 4:** Risk classification analysis of predicting cardiac death for adding GLS to anthropometric factors and blood examination parameters.

Cardiac death (-)		Predicting risk from age, sex, BNP, Na, Cre, Hb, LVEF and GLS				
Predicting risk from age, sex, BNP, Na, Cre, Hb and LVEF		0–0.0111	0.0111–0.0196	0.0196–0.0376	0.0376–1	% reclassified
	0–0.111	249	5	0	0	2
	0.0111–0.0196	12	237	6	0	7
	0.0196–0.0376	0	16	227	6	9
	0.0376–1	0	0	7	221	3
Cardiac death (+)		Predicting risk from age, sex, BNP, Na, Cre, Hb, LVEF and GLS				
Predicting risk from age, sex, BNP, Na, Cre, Hb and LVEF		0–0.0111	0.0111–0.0196	0.0196–0.0376	0.0376–1	% reclassified
	0–0.0111	3	0	0	0	0
	0.0111–0.0196	0	1	1	1	50
	0.0196–0.0376	0	0	8	0	0
	0.0376–1	0	0	0	29	0
Continuous NRI = 0.139 (95%CI 0.170–0.447), p = 0.377						

BNP, B-type natriuretic peptide; CI, confidence interval; Cre, creatinine; GLS, global longitudinal strain; Hb, hemoglobin; LVEF, left ventricular ejection fraction; Na, sodium; NRI, net reclassification improvement.

**Table 5:** Risk classification analysis of predicting MACEs for adding GLS to anthropometric factors and blood examination parameters.

MACEs (-)		Predicting risk from age, sex, BNP, Na, Cre, Hb, LVEF and GLS				
Predicting risk from age, sex, BNP, Na, Cre, Hb and LVEF		0–0.0426	0.0426–0.0699	0.0699–0.122	0.122–1	% reclassified
	0–0.0426	211	33	7	0	16
	0.0426–0.0699	87	120	32	2	50
	0.0699–0.122	1	75	132	30	45
	0.122–1	0	2	33	156	18
MACEs (+)		Predicting risk from age, sex, BNP, Na, Cre, Hb, LVEF and GLS				
Predicting risk from age, sex, BNP, Na, Cre, Hb and LVEF		0–0.0426	0.0426–0.0699	0.0699–0.122	0.122–1	% reclassified
	0–0.0426	3	3	0	0	50
	0.0426–0.0699	2	11	2	1	31
	0.0699–0.122	0	2	15	2	21
	0.122–1	0	0	4	62	6
Continuous NRI = 0.364 (95%CI 0.167–0.562), p<0.001						

BNP, B-type natriuretic peptide; CI, confidence interval; Cre, creatinine; GLS, global longitudinal strain; Hb, hemoglobin; LVEF, left ventricular ejection fraction; MACEs, major adverse cardiovascular events; Na, sodium; NRI, net reclassification improvement.

### Reproducibility

Variability of GLS measurements using the same images in 10 patients was 0%. The time required for GLS analysis was 21±3 seconds. Bias ± SD of GLS assessed by another apical 3 images that were acquired by two different sonographers during the same examinations was 0.1 ± 1.1% (p = 0.7022).

## Discussion

The major findings of this study are: (1) GLS cut-off values of 7% and 14% obtained from fully automated software stratified the risk of future CD in patients with known or suspected HF; (2) when GLS cut-off values of 7% and 14% and BNP cut-off values of BNP of 100 pg/mL and 200 pg/mL were employed, patients were effectively stratified from low to high risk for future MACEs; (3) automated GLS measurements were significantly and incrementally more powerful than anthropometric factors and blood examination parameters including BNP for predicting future outcomes.

### Previous studies

Echocardiography is a versatile technique to provide useful information regarding cardiac structure and function. Under current guidelines, echocardiography is recommended in the diagnostic workup of HF patients to diagnose HFpEF, HFmrEF, and HFrEF [4]. Although three-dimensional TTE provides accurate and reliable measurements of LVEF [16], 2D TTE determined LVEF remains the most widely used technique in clinical practice and currently guides both diagnosis and therapy in HF patients [17]. However, LVEF calculation usually requires manual tracing of LV contours. This introduces experience-dependent observer variability, and it is time consuming [9, 18]. Previous studies have demonstrated that LVEF does not accurately discriminate the risk of cardiovascular outcome in HFpEF patients, although LVEF is known to be an independent predictor of cardiac mortality [19, 20].

Myocardial strain based on 2D speckle tracking analysis has been used for objective and reliable assessments of LV function. GLS has proven beneficial in detecting impaired systolic function in HFpEF patients who manifest normal LVEF [21, 22]. GLS has also been demonstrated as a superior predictor of mortality in HFrEF patients, compared with LVEF [8]. Moreover, it has emerged as a powerful predictor for cardiovascular death and hospitalization in HFpEF patients [6, 7, 23]. Although GLS is less experience-dependent and more reproducible than conventional LVEF measurements, there are still sources of measurement variation that are significant [9, 18]. Adoption of fully automated 2D speckle tracking software can solve this problem.

BNP is a potential biomarker for diagnosis and prognostic stratification for HF patients [11, 17, 24]. Although some studies classify HF patients by using BNP and LVEF to predict future cardiac events [12, 13], it remains unclear whether both GLS measured by automated approach and BNP can stratify HF patients by their likelihood of future cardiac events.

### Current study

To the best of our knowledge, this is the first study to predict future cardiac events from GLS, using fully automated 2D speckle tracking analysis and BNP levels. In this study, fully automated software erroneously determined LV endocardial border in approximately 5% of patients; thus, the feasibility of measuring LV volumes, LVEF, and GLS was 95%. It allows 100% reproducibility of GLS measurements as long as the same images are used for analysis and test-retest variability using another image was very low.

In this study, univariate Cox proportional hazard analysis revealed that both LVEF and GLS were significant predictors of CD and MACEs, which aligns with previous studies [8, 25, 26]. Survival CART analysis, including BNP, LVEDVI, LVEF, and GLS, revealed that GLS is the most useful predictor of CD, and that GLS and BNP are the most useful for predicting MACEs, whereas LVEF is not a discriminator of either CD or MACEs. This means that GLS is a better prognosticator than LVEF in patients with known or suspected HF. Using results from the derivation cohort, we approximated cut-off values of GLS as 7% and 14% for CD. We also approximated cut-off values of BNP as 100 pg/mL and 200 pg/mL. These cut-off values worked well in the validation cohort. They are easily remembered, and should prove very useful for management of HF, because both determinations are fully automated.

### Clinical competencies

Predicting future cardiac events in patients with known or suspected HF is important, but conventional measurements of LVEF and GLS require examiner expertise and are subject to observer variability [9, 18]. GLS measurements using fully automated 2D speckle tracking analysis software have high feasibility without observer variability. Cut-off values of BNP and GLS values are easily remembered; thus, they are very useful for predicting future cardiac events in busy routine practice.

### Study limitations

Several limitations of this study should be considered. First, there is no gold standard to determine measurement accuracy of GLS. Second, 40% of patients were asymptomatic. However, no symptoms precluded the occurrence of HF. Inclusion of asymptomatic patients allows our study results to be more generalizable. Third, we did not perform subgroup analysis in HFpEF, HFmrEF, or HFrEF because the event rate of HFpEF was very low. Finally, this study was a retrospective observational study in a single center in Japan. Thus, validation studies in other centers and countries should be performed.

## Conclusions

We validated prognostication using fully automated GLS and BNP in patients with known or suspected HF. Since this is a fully automated approach, it does not suffer from observer variability. Our results facilitate adoption of GLS measurements using fully automated 2D speckle tracking software in everyday practice.

## Supporting information

S1 Data (XLSX)
